# Costs of Inaction on Maternal Mortality: Qualitative Evidence of the Impacts of Maternal Deaths on Living Children in Tanzania

**DOI:** 10.1371/journal.pone.0071674

**Published:** 2013-08-19

**Authors:** Alicia Ely Yamin, Vanessa M. Boulanger, Kathryn L. Falb, Jane Shuma, Jennifer Leaning

**Affiliations:** 1 Global Health and Population, Harvard School of Public Health, Boston, Massachusetts, United States of America; 2 Program on the Health Rights of Women and Children, Franςois-Xavier Bagnoud Center for Health and Human Rights, Harvard University, Boston, Massachusetts, United States of America; 3 Chronic Disease Epidemiology, Yale School of Public Health, New Haven, Connecticut, United States of America; 4 Program on the Health Rights of Women and Children, Franςois-Xavier Bagnoud Center for Health and Human Rights, Harvard University, Dar es Salaam, Tanzania; 5 François-Xavier Bagnoud Center for Health and Human Rights, Harvard University, Boston, Massachusetts, United States of America; Middlesex University London, United Kingdom

## Abstract

**Background:**

Little is known about the interconnectedness of maternal deaths and impacts on children, beyond infants, or the mechanisms through which this interconnectedness is established. A study was conducted in rural Tanzania to provide qualitative insight regarding how maternal mortality affects index as well as other living children and to identify shared structural and social factors that foster high levels of maternal mortality and child vulnerabilities.

**Methods and Findings:**

Adult family members of women who died due to maternal causes (N = 45) and key stakeholders (N = 35) participated in in-depth interviews. Twelve focus group discussions were also conducted (N = 83) among community leaders in three rural regions of Tanzania. Findings highlight the widespread impact of a woman’s death on her children’s health, education, and economic status, and, by inference, the roles that women play within their families in rural Tanzanian communities.

**Conclusions:**

The full costs of failing to address preventable maternal mortality include intergenerational impacts on the nutritional status, health, and education of children, as well as the economic capacity of families. When setting priorities in a resource-poor, high maternal mortality country, such as Tanzania, the far-reaching effects that reducing maternal deaths can have on families and communities, as well as women’s own lives, should be considered.

## Introduction

Globally, an estimated 287,000 women die each year due to maternal causes [1], despite maternal mortality being an overwhelmingly preventable cause of death [2]. Women in sub-Saharan Africa are disproportionally at risk of maternal death, including those in Tanzania where the estimated maternal mortality ratio (MMR) in 2010 stood at 454 deaths per 100,000 live births [1], [3]. A woman dies almost every hour from maternal causes in Tanzania [1]. These figures alone, however, fail to capture the far-reaching repercussions of these maternal deaths on a woman’s family and children.

Maternal death in Tanzania, as elsewhere, is most often the end result of compounded discrimination and deprivations that women face across their lives, which affect their health and human rights more broadly [4]. For instance, low educational attainment for women has been associated with reduced reproductive autonomy regarding family planning utilization and increased parity [5], and has been directly linked to increased risk of maternal mortality as it relates to reduced knowledge of obstetric warning signs [6]. Low status of women within partnerships and extended families is reflected in the lack of women’s decision-making power, including their ability to seek care during pregnancy [7], or to choose a facility-based delivery [8]. Poverty intersects with gender subordination, as the lack of financial means for transport and health care fees has been found to be a key barrier to receiving obstetric care in Tanzania [7]–[9]. Such economic barriers are exacerbated for women who have limited economic opportunities and control over household financial resources, even when their lives hang in the balance.

Undeniably, the presence of a mother is a critical protective factor for the survival of young children, particularly in terms of breastfeeding or accessing healthcare, even though extended families and communities attempt to support the children of mothers who die in a range of contextually-specific ways [10]. Most maternal deaths, however, occur in communities of desperate poverty, where the precariousness of life conditions for everyone often impedes robust community-level support initiatives.

### Background

Previous quantitative research has documented a heightened risk of infant mortality among those index children whose mother had died [11], likely due to absence of breastfeeding [12], [13] or complications from delivery [14]. For example, 53% of all perinatal deaths were found to be related to labor complications in rural Kenya [14]. However, the effects of a mother’s death can also reverberate across childhood, as demonstrated among a cohort in Bangladesh, where only 24% of maternal orphans survived until age ten, compared to 89% of children whose mother was still alive [12]. Another study on the impact of a mother’s death conducted in Haiti, found a 55% increased chance of death for children under 12 years of age [15]. Despite stark indications from these few studies, remarkably little is known about the mechanisms by which maternal mortality undermines the health and development of older children and adolescents and exacerbates psychosocial vulnerabilities in already impoverished environments, such as those found in rural Tanzania. Apart from the survival of young children and infants, the impact of maternal death on older children has only been partially addressed through literature on the health and psychosocial status of HIV/AIDS orphans. These children have been found to be at risk of an array of negative outcomes, including lower educational attainment, substance abuse, poor mental health outcomes, and increased sexual risk, while there has been mixed evidence on the association between orphanhood and malnutrition [16]–[20]. Many studies have not explored the life course impacts of a maternal death and therefore inadequately assess for potential intergenerational effects of maternal mortality that could be transmitted through earlier marriages, younger age at first birth for surviving female children, and lower household wealth, for example, which thereby perpetuate cycles of poverty and maternal mortality within the family and the community.

Given the need to understand the interconnectedness between maternal deaths, child outcomes, and family impacts, as well as the mechanisms through which they are related, the objectives of this qualitative study were threefold: (1) to provide qualitative insight regarding how maternal mortality affects child and family well-being; (2) to identify common structural and social factors that lead to high rates of maternal mortality and child vulnerabilities; and (3) to propose policy and program directions for the improvement of maternal and child health in Tanzania, highlighting the consequences and far-reaching costs of inaction. Qualitative evidence is needed to explore differential effects by gender after a maternal death has occurred and to provide a rich, context-specific narrative to guide future research and inform policies and programs aimed at supporting maternal orphans and other vulnerable children throughout their development.

## Methods

### Ethics Statement

Study protocols were approved by the Harvard School of Public Health Institutional Review Board and the National Institute of Medical Research in Tanzania.

Informed consent was read verbatim by the research coordinator and all participants indicated consent through either a signature or thumb print.

### Study Design

The present analysis focuses on a sample of emerging themes from qualitative research conducted in Tanzania, which is part of an ongoing four-country mixed methods study (Tanzania, Ethiopia, Malawi, and South Africa) on the impacts of maternal deaths on living children. Qualitative data collection is currently ongoing in the other countries, thus, in this paper we only present data from Tanzania to provide a rich, context-specific narrative of the impacts of maternal deaths on living children. The overarching study will also include quantitative estimates of elevated child mortality and related outcomes and associations with maternal mortality utilizing longitudinal Demographic Surveillance Site (DSS) data in Tanzania and Ethiopia; analyses are currently underway. Three regions in Tanzania (Rufiji, Kilombero, and Ulanga) were selected for inclusion into the study, based on having high regional rates of maternal mortality, a diversity of ethnicities and religions, and alignment with DSS coverage for the quantitative component of the parent study. Key stakeholders based at national organizations or orphanages were also interviewed in Dar es Salaam (N = 21). In-depth interviews included guardians of orphaned children and adult family members of women who died due to maternal causes (N = 45), in addition to local stakeholders including community development officials, social workers, community health workers, etc. (N = 14). Twelve sex-stratified focus groups were also conducted among 83 participants, which included local leaders such as teachers and religious advisors. Adult family members were identified and recruited through partnerships with local health facilities, civil society organizations, or through snowball sampling. A Tanzanian research coordinator also worked in partnership with community health workers to confirm that female deaths were due to maternal causes through an examination of medical records. Stakeholder key informants and focus group participants were identified via local community leaders, a desktop review of existing programs, and snowball sampling. Data collection occurred between April 2012 and April 2013.

Semi-structured in-depth interviews were conducted via research staff with English-Kiswahili translation performed by the research coordinator. All in-depth interviews were conducted in a private location of the participant’s choosing and were digitally recorded. Interviews with adult family members included topics within three key areas: (1) general characteristics of the family to provide socioeconomic context; (2) circumstances that led to maternal mortality and the impacts on the children and family; and (3) availability and accessibility of services for maternal orphans. In the case of multiple maternal orphans in a family, the impacts of the mother’s death on each child were discussed for each respective orphan. Focus group topics included general community perceptions of orphans and services available. Participants were also probed about community perceptions of maternal orphans at different ages, including infants and young children, school age children, and adolescents, and how the impacts of a maternal death may manifest differently in boy and girl children. Focus group participants were also asked about how the community provides informal social support, as well as the availability of formal government services for orphans. Stakeholder interviews included topics such as availability of programs for orphans and challenges for implementing such programs and policies, including financial issues and political will for such programs. Each in-depth interview and focus group took between 1.5 and two hours to complete. Family member and focus group participants also received 10,000 Tanzanian Shillings ($6.25 USD) for their participation.

All interviews and focus group discussions were transcribed from the digital recording and translated into English by the research coordinator. All digital recordings were erased upon transcription. Utilizing an inductive approach to coding, two research staff then coded the transcripts, discussing and editing themes as they emerged. All analyses were conducted in NVivo 10.

## Results

### Background Characteristics of Guardians, Key Informants, and the Women Who Died from Maternal Causes

Forty-five key informant interviews were conducted in Rufiji, Kilombero, and Ulanga with guardians of maternal orphans. Twenty-six percent of the interviews were with husbands of the deceased, 24% were with a sister of the deceased, and 20% were with a grandmother of the orphan. Stakeholder interviews were conducted with medical personnel, district health administrators, heads of orphanages, and representatives from non-governmental organizations (N = 14) and with representatives at national organizations in Dar es Salaam (N = 21). Focus group participants were a mix of teachers, religious leaders, community health workers, village chairs, small business owners, and leaders of local women’s groups.

This specific analysis focuses on the effects of a maternal death on orphans, families, and communities in rural Tanzania looking specifically at the far reaching, interconnected consequences of a maternal death on family dissolution, guardianship, socio-economics, health, nutrition, education, and community programming for maternal orphans. Drawing from the key themes of the semi-structured interview guide, we analyzed the qualitative findings looking for disparities among orphans by age and gender, as well as issues relating to access to education and healthcare, and psycho-social outcomes.

Of our study population, most maternal deaths occurred amongst women in formal or informal unions (71%), 18–35 years old, with a standard 7 level of education (US equivalent of completion of primary school) (54%). None of the women who experienced maternal deaths had any secondary education (Table 1).

**Table 1:** Characteristics of women who died due to maternal causes (*n = 41*).

	*n (%)*
**Age at death (years)**	
15–18	2 (4.9)
19–24	6 (14.6)
25–29	6 (14.6)
30–35	6 (14.6)
35+	4 (9.8)
Missing values	17 (41.5)
**Level of Education**	
None	5 (12.2)
Less than Standard 7	6 (14.6)
Standard 7	22 (53.7)
Secondary	0 (0)
Missing values	8 (19.5)
**Marital Status**	
Single	3 (7.3)
Divorced/Separated	5 (12.2)
Married	22 (53.6)
Partnered	7 (17.1)
Missing values	4 (9.8)
**Prior birth(s)**	
0	5 (12.2)
1	6 (14.6)
2	5 (12.2)
3	9 (21.9)
4	5 (12.2)
≥5	8 (19.5)
Missing values	3 (7.3)

### Implications of a Maternal Death on Maternal Orphans

#### Health Care

Tanzania’s National Health Policy provides free care for pregnant women and children under five [21] at government run facilities, though in practice it is common for both women and children to face fees when seeking care. Many informants in our sample (27%) mentioned their struggle to pay for indirect costs associated with delivering in a facility, including transportation, fuel, and ambulance costs, and the financial and logistical arrangements of residing near a hospital in the weeks leading up to delivery, as a significant barrier to seeking care. Substantial out-of-pocket payments for child health care also affected the families in our sample; direct health care fees and additional ‘unofficial’ costs associated with medicine, medical treatment, and supplies, particularly for recurring or chronic health conditions, were frequently cited as posing a major obstacle to accessing care. As one stakeholder explained:

‘From 0–7 years, they [orphans] face frequent diseases. Although the government policy says all children under 5 get free health care, that is not the case because the doctor will prescribe medication which the family will have to go and buy. So the burden of hospital costs will fall to the guardian.’

Delays in seeking care for these reasons often exacerbate health conditions and place children, especially orphaned children who may not be given priority within the family, at increased risk of serious morbidity and mortality [15].

#### Nutrition

The majority of maternal orphans are not breastfed; in our sample only 14.6% (6 infants) were ever breastfed and 4.9% (2 infants) were breastfed for longer than one month. As was mentioned in focus group discussions in each of the three districts, the cost of formula is too high for nearly all families, further straining household budgets. As a result, maternal orphans are given cow’s milk instead, which often causes gastric distress. As one male focus group participant noted:

‘That [orphaned] child may lack some milk during his/her development stage [because it is too] expensive to purchase and so some families opt for cow milk. Further, if children do not get the right amount of formula at their age, it may lead them into not growing up well and may [even] lead to death.’

Depending on how children are prioritized within the home, orphaned children may be at higher risk of under nutrition and increased limitation in terms of food quantity and quality. Under nutrition in infancy is especially critical, as it can result in stunting [22].

While nutrition is essential for health, it is also important for educational performance. Mothers’ roles in rural Tanzania include ensuring that meals are prepared for their children, and, in their absence, guardians and teachers who participated in our focus groups explained that orphaned children will often return home from school during lunch break to find no food. If they return to school without having eaten, teachers report a noticeable decrease in concentration and attention span. As one female focus group participant explained,

‘Children who have lost mothers, their level of education is low. For instance, in the afternoon children usually go home for lunch…if there is no mother to cook and give children food, they will come back to school hungry and will not be able to concentrate…’

#### Education

A common theme in our research findings was the important role mothers have in prioritizing and supervising education within the family. As stated by one father,

‘Whenever their mother was around she would make sure that [the] kids stay in the house when they are home from school. She would help them with school work and so forth. Right now when they come back from school, they just go wander around [the neighborhood]. And I think the school progress of my first-born girl has gone down because my wife is not around to help her with school homework.’

Decreased concentration and lower educational performance following a mother’s death and the need for supervision and assistance in order to complete homework and other school-related tasks were mentioned by focus group participants in each of the three districts in our study as a consistent struggle for guardians of orphaned children. In addition, focus group participants from each district cited the financial burden of paying for school fees, contributions, and supplies as a significant challenge, which in some cases results in delays in the timing and completion of school for orphaned children.

#### Exacerbated risks for girl orphans

The underlying power structures and gender inequalities that drive high maternal mortality are also reflected in the differential impacts on the children who are left behind after a maternal death [23]. At the household level, girl children are often responsible for caring for younger children and are pressured to complete household chores, which often results in significant interruptions in schooling and early school drop-outs [24]. For instance, one stakeholder explained,

‘For girls when they reach an age where they are able to work it really affect[s] them in their performance at school because most of the time they will be doing home activities – washing dishes, cooking for their siblings etc. So a girl child becomes like a mother.’

Early marriage was a common theme in our study sample for both boy and girl orphans. Early school drop-out and an increase in household or financial responsibilities may lead orphans to engage in early marriage in order to find a partner to support and care for them, so that they are relieved from struggling on their own. Focus groups, stakeholders, and key informants identified the correlation between cultural norms that support early marriage for girl orphans and early sexual debut and pregnancy.

Further, consistent with other studies, our findings suggest that less care and support from a guardian, lower educational attainment, and fewer economic opportunities for girls may result in increased engagement in high-risk sexual behaviors, particularly transactional sex, in order to meet basic needs [25]. As one stakeholder stated,

‘In most cases when a girl gets pregnant she is all by herself [and] sometimes she doesn’t know who the father is, so she thinks that in order to keep [and care for] this child, a solution is to go out with another man. In the end, she will become pregnant again. Eventually, she finds that she has 6 children who belong to different fathers and no one [father] is responsible for them…so one problem multiplies into another.’

### Implications of a Maternal Death on the Family

#### Family dissolution

After a maternal death in Tanzania, it is customary for families to hold a meeting with extended family members, where critical decisions are made about the care, placement, and guardianship of orphaned children. Depending on the age of the orphan, the biological father, and the number of children in the family, it is common for siblings to be separated and placed in different homes, most often under the care of a maternal aunt or female elder. Thus, children who are grieving the loss of their mother are often immediately forced to transition away from their home, their father, and their siblings. The resulting level of care that the orphan receives is relatively arbitrary and dependent upon the level of involvement from the new guardian, as well as the family’s resources.

#### The role of the father and guardian

While some fathers do continue to care for their biological children, our data indicated that fathers rarely care for non-biological children of the deceased who share the same household. As was explained in a focus group discussion, after a maternal death, if a woman has children with multiple partners,

‘…usually each father will come and take their [own] children and look after them because this [male] partner who is left behind, will be like a step father to these [other] children and he is not willing to take care of another man’s child after his wife has passed on.’

Moreover, in rural Tanzania, it is not common for fathers to offer sustained financial support for their children when they are not one of the primary caretakers. Stakeholders asserted that the erosion of collective social support systems, as was emphasized in Tanzania under Julius Nyerere’s ‘Ujamaa’ immediately after the country achieved independence [26], has left fathers disinclined to assume individual responsibility for the care and support of their children, especially infants.

As is supported in the literature, when a father does continue to care for his children, our findings suggest it is likely that he will remarry [27] in order to have help in the home. Additionally, our findings indicate that though some maternal orphans are cared for equally, as though they are biologically related, others face differential treatment in the home depending on the quality of care provided by the guardian. Households in rural Tanzania operate under extremely precarious socio-economic circumstances and a scarcity of basic resources, forcing families to make difficult decisions regarding resource-allocation within the home, which are often determined by gender and other social norms. As one male focus group participant noted:

‘Men also contribute so much into bringing impact to the children because once they remarry, the new woman would want to bear her own children and she would want her children to be given a priority…as a result the man would take his prior children to go and live with other relatives and those children may end up suffering.’

#### Economic Dtrains

Among many other impacts, the loss of a mother has many economic consequences for a family, including the loss of household and field labor and perhaps the loss of a secondary income. This was a particular challenge among interviewees as the majority of families in Rufiji, Kilombero, and Ulanga (71%) earned their livelihoods through subsistence farming and selling the meager surplus crops they had.

In addition, a maternal death removes the crucial unremunerated care-giving and other household work that women perform, which is essential to a functioning household [28]. Our findings corroborate that when orphaned children are dispersed among family members or when a father remarries and orphans are combined with children from the new union, increased household sizes further strain already limited household resources and finances. Fewer financial resources are available for each individual child for educational and health care needs, clothing, and food. Thirty-four percent of the guardians that we interviewed (6 male, 8 female) said that although they did not have the expendable household income to support additional children, they felt they had no choice but to try to care for the orphaned children since there was no viable alternative.

#### Support services and programs for maternal orphans and other vulnerable children

Only 14.6% of the key informants in our study received any support though government, NGO, or community programs for orphans and most vulnerable children (MVCs) and just 27% of the key informants were even aware of programs that provide support for MVCs.

Local and national stakeholders in our study described the programs available to support maternal orphans and other MVCs as being limited, fragmented, underfunded, and often of poor quality in Tanzania. For example, the expansion of Most Vulnerable Children Programs to every district throughout Tanzania, a key mechanism of the National Plan of Action for Most Vulnerable Children, only covered 5.85% of Tanzanian villages by the end of 2011 [29]. Further, stakeholders in our sample also described a lack of coordination, not only among different ministries, such as the Ministry of Health and Social Welfare and the Ministry of Community Development, but also between national and district level government programs, whereby the former is responsible for guidelines and the latter for budgeting and implementation. Other challenges include a shortage of human and financial resources for implementation of MVC programs. Focus groups suggested that at the community level this lack of programming translates into health facilities being unable to provide infant formula or other nutritional support to maternal orphans and schools being unable to provide uniforms and supplies.

## Discussion

Findings from this study document the profound effects of a maternal death on child health and family well-being. Our qualitative findings contextualize quantitative studies that have documented the staggeringly negative impacts of a mother’s death on children, and offer pathways through which the two are related [12], [15]. Common challenges faced by extended families included family dissolution and economic strains. Consistent with other studies, most caregivers in our sample were women, thus challenges to orphaned children’s health and well-being may be compounded as female caregivers often do not have sufficient control over household financial resources to ensure appropriate support for orphans [30]. Nutritional challenges were cited by over a third (34%) of the caregivers in our sample, with especially acute consequences among infants as the cost of formula was prohibitively high for families. Children were also faced with additional challenges for educational attainment and access to timely health care in the already impoverished and precarious environments found in rural Tanzania. Future studies should assess whether certain programs, such as conditional cash transfers, payments for school fees and associated costs, etc. are able to mitigate these observed challenges among maternal orphans [31]–[33].

Our study demonstrates that gender inequalities that affect women’s health across their lifespans and drive high levels of maternal mortality also affect maternal orphans. Within families in our study, for instance, many women who died due to maternal causes were at high parity (54% had 3 or more children). Indeed, within rural regions of Tanzania, the total fertility rate is 6.1 children and women at the lowest socioeconomic levels with no education have the highest fertility rates [3]. Therefore, efforts to improve reproductive autonomy and contraceptive use would reduce the number of pregnancies and maternal deaths due to pregnancy complications [34] and ultimately promote well-being among living children. As one stakeholder stated:

‘In the community, there should be [a] sustainable program that can first empower women … For instance she should have her own money and power to decide when to go to the hospital when she gets a problem. I hope this will minimize orphans because other women die because of delay to reach to the health facility.’

In addition, social norms that prescribe rigid gender roles and de-emphasize fathers’ roles in child rearing [26] exacerbate the potential effect of a mother’s death on a child’s life, as fathers often do not take responsibility for guardianship of their own children after a maternal death. These norms also contribute to family dissolution, as orphans are commonly separated among other female care-takers in the extended family.

Few families (27%) were aware of services or programs for orphans, in which they may be eligible. In addition, existing programs do not systematically address the urgent nutritional needs of infants who survive after a maternal death, including the prohibitive costs of formula, which thus places maternal orphans at a higher risk of under nutrition, stunting [35], and poor development during this critical period [36]. Stakeholders also detailed the lack of human and financial resources available to successfully implement programs in rural villages, in addition to challenges related to sustainable budgeting, as Tanzania does not have a sufficient budget to provide support for most vulnerable children. Coordination across sectors, including health, community development, and education, as well as across levels of government is urgently needed to create a comprehensive response to families affected by maternal mortality.

These findings should be interpreted with limitations in mind. Although the study provides in-depth qualitative insight into the mechanisms linking maternal mortality and child outcomes, the linkages cannot be assumed to be causal and the results cannot be generalized beyond the study population. In addition, women whose deaths were never recorded or known by community health workers are not included in this sample; experiences of their children may be different than those included in the study.

Despite these limitations, the study illuminates the high costs to surviving children and their families of failing to reduce maternal mortality in this region and highlights potential pathways through which maternal mortality and maternal orphan morbidities are linked. Our findings are consistent with the existing literature on vulnerable children, but highlight the specific health and social impacts that a maternal death can have throughout the course of a child’s life and the all too frequent cycle of poverty and suffering that stems from the high cost of failing to prevent a maternal death and subsequent inaction to protect and support maternal orphans. Efforts to mitigate these negative outcomes should include the implementation of a comprehensive, multi-sectoral approach to providing care for maternal orphans and other MVCs, as called for in Tanzania’s National Costed Plan of Action 2, and for a more effective, efficient, and sustainable child protection system in general [37]. This study in rural Tanzania provides a fresh empirical and intergenerational perspective supporting what is already known about the underlying factors that make maternal mortality a persistent issue in areas where inequitable gender and social norms undermine the status of women [38]. Until these root causes are addressed, women will continue to die in pregnancy and childbirth and the children and families that are left behind will remain in a cycle of poverty and suffering.
